# Outcomes After SARS-CoV-2 Vaccination Among Children With a History of Multisystem Inflammatory Syndrome

**DOI:** 10.1001/jamanetworkopen.2022.4750

**Published:** 2022-03-28

**Authors:** Matthew Wisniewski, Angela Chun, Stefano Volpi, Eyal Muscal, S. Kristen Sexson Tejtel, Flor Munoz, Tiphanie P. Vogel

**Affiliations:** 1 Division of Critical Care Medicine, Department of Pediatrics, Baylor College of Medicine and Texas Children’s Hospital, Houston; 2 Division of Rheumatology, Department of Pediatrics, Baylor College of Medicine and Texas Children’s Hospital, Houston; 3 U.O.C. Clinica Pediatrica e Reumatologia, Centro per le malattie Autoinfiammatorie e Immunodeficienze, Istituto Giannina Gaslini and DINOGMI, Università degli Studi di Genova, Genova, Italy; 4 Division of Cardiology, Department of Pediatrics, Baylor College of Medicine and Texas Children’s Hospital, Houston; 5 Division of Infectious Diseases, Department of Pediatrics and Department of Molecular Virology and Microbiology, Baylor College of Medicine and Texas Children’s Hospital, Houston

## Abstract

This case series evaluates the outcomes following COVID-19 vaccination in patients previously diagnosed with multisystem inflammatory syndrome in children at children’s hospitals in Texas and Italy.

## Introduction

Most children who contract SARS-CoV-2 are asymptomatic or mildly symptomatic.^1^ However, a subset of children subsequently develop a severe hyperinflammatory condition called multisystem inflammatory syndrome in children (MIS-C) 4 to 6 weeks after having COVID-19.^2^ The underlying mechanisms of MIS-C remain unclear,^3^ leading to hesitation to vaccinate children with a history of MIS-C against SARS-CoV-2 because of concerns for a reoccurrence of hyperinflammation. In December 2020, the US Food and Drug Administration and the Italian Drug Agency (Agenzia Italiana del Farmaco) provided emergency use authorization for the COVID-19 vaccine, BNT162b2, in individuals 16 years or older. In May 2021, the vaccine became available to individuals 12 years or older. We aimed to evaluate outcomes following SARS-CoV-2 vaccination in patients previously diagnosed with MIS-C and hypothesized that vaccination would be well-tolerated.

## Methods

This case series was approved by the institutional review boards at Baylor College of Medicine in Houston, Texas, and the Regione Liguria Ethics Committee in Genova, Italy. Informed consent was waived because this was a minimum risk study. The reporting guideline for case series was used for this study; a case series was selected because of the rare occurrence of MIS-C and formed by retrospective review of medical records. We included the patient self-reported race (Asian, Black, and White) and ethnicity (Hispanic and non-Hispanic) recorded in the electronic record. Race and ethnicity were included in our study because of the increased incidence of MIS-C in racial and ethnic minority populations.

We included patients who presented at Texas Children’s Hospital in Houston, and Gaslini Children’s Hospital in Genova, Italy, from April 2020 to June 2021 with an acute febrile illness that fulfilled the US Centers for Disease Control and Prevention MIS-C case definition.^4^ During this time, 169 patients were diagnosed at Texas Children's Hospital, and 56 of those patients were eligible for vaccination (23 patients aged 16 years or older; 33 patients aged 12 to 15 years); 24 patients were diagnosed at Gaslini Children’s Hospital, and 7 were eligible for vaccination (2 patients aged 16 years or older; 5 patients aged 12 to 15 years). As part of a subspecialty follow-up after hospitalization, patients with a history of MIS-C were counseled and encouraged to receive COVID-19 vaccination 90 days or more after diagnosis. In total, 15 of 63 eligible patients (24%) were vaccinated. Vaccinated patients were formally queried regarding vaccine reactogenicity and recurrence of hyperinflammation. No statistical testing was performed, and findings are descriptive only. Data were managed in Excel (Microsoft Office).

## Results

The mean (range) age of vaccinated MIS-C patients was 14.4 (12-18) years; 10 patients (67%) were male, and 10 (67%) were from racial and ethnic minority groups (ie, Asian patients, Black patients, and Hispanic patients) (Table). Most patients were previously healthy, except 4 (27%) with asthma and 1 (7%) with a congenital heart defect. All 15 had positive SARS-CoV-2 testing: 13 (87%) had spike immunoglobulin G (IgG) at presentation, and 2 (13%), for whom serology was unavailable at the time of MIS-C, had COVID-19 confirmed by nucleic acid testing 4 to 6 weeks prior to MIS-C. Cardiac involvement, defined by troponin elevation or echocardiographic or electrocardiographic changes as outlined in the Brighton Collaboration case definition,^5^ occurred in 14 patients (93%). Intensive care was required for 10 patients (73%) after 11 patients (73%) presented in shock; vasoactive support was needed for 5 patients (33%) and invasive mechanical ventilation was needed for 2 patients (13%). Treatment included corticosteroids for 15 patients (100%), 11 patients (73%) received high dose immunoglobulin, and 10 patients (67%) received additional immunomodulation (anakinra). The mean (range) hospital length of stay was 10.9 (2-33) days. At the last outpatient cardiac evaluation, all patients had normal cardiac function without coronary dilation.

**Table.** Demographics, Disease Severity, and Vaccine Reactogenicity

Demographics	Participants, No. (%) (N = 63)	
	Vaccinated (n = 15)	Not vaccinated (n = 48)
Age, mean (range), y	14.4 (12-18)	15.3 (12-21)
Male	10 (67)	27 (56)
Female	5 (33)	21 (44)
BMI, mean (range)	25.1 (18.9-44.8)	26.5 (16.7-48.8)
Racial or ethnic minority group^a^	10 (67)	39 (81)
Disease severity		
Intensive care	10 (73)	33 (69)
Hypotension	11 (73)	33 (69)
Ionotropic support	5 (33)	27 (56)
Intubation	2 (13)	11 (23)
Received corticosteroids	15 (100)	48 (100)
Received high dose immunoglobulin	11 (73)	34 (71)
Received anakinra	10 (67)	37 (77)
Length of stay, mean (range), d	10.9 (2-33)	11.4 (3-55)
Vaccine reactogenicity (n = 12)^b^		
Local site		
Pain	7 (58)	NA
Redness	0	NA
Swelling	0	NA
Systemic		NA
Fatigue	4 (33)	NA
Headache	4 (33)	NA
Myalgia	0	NA
Fever	1 (8)	NA
Nausea	1 (8)	NA

Abbreviations: BMI indicates body mass index, which is calculated as weight in kilograms divided by height in meters squared; NA, not applicable.

^a^ For this study, racial and ethnic minority groups include Asian patients, Black patients, and Hispanic patients.

^b^ Three Patients did not directly report.

Vaccination occurred at a mean (range) of 189.5 (91-340) days from MIS-C presentation (Figure). At submission, a mean (range) of 181.3 (56-286) days had elapsed since the patients completed their last vaccine. Twelve patients were directly queried for vaccine reactogenicity, and data for 3 patients were available by review of the medical record. No major adverse events were reported following vaccination. In addition, no patients have developed a recurrence of MIS-C or any hyperinflammatory condition.

**Figure.**
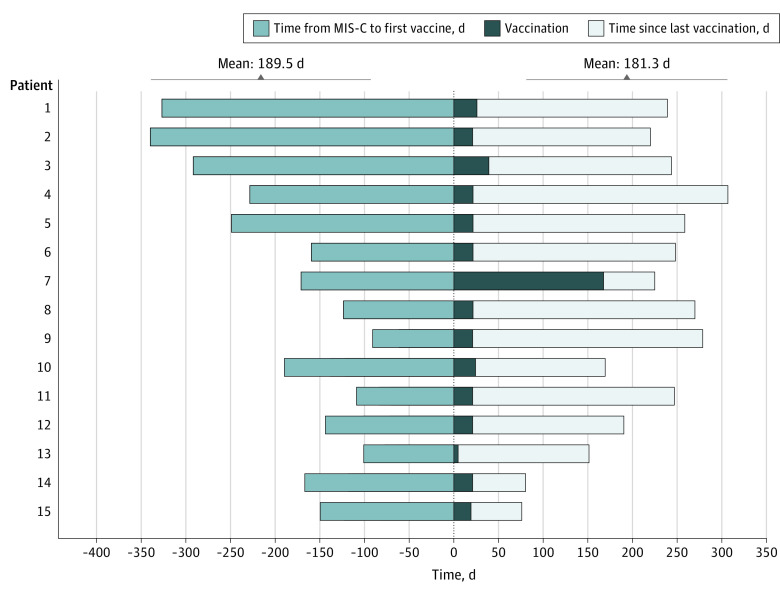
Time Courses of Patients With Multisystem Inflammatory Syndrome in Children (MIS-C) Receiving SARS-CoV-2 Vaccination Fifteen patients (5 girls and 10 boys, aged 12 to 18 years) with a history of MIS-C were vaccinated against SARS-CoV-2. Most patients received 2 doses, 21 days apart. One patient received the 2 doses 6 months apart, which was suggested as a possible immunization schedule by Italian authorities for patients with a history of COVID-19 in the preceding 12 months. The parents of another patient elected in advance to follow a personalized vaccination strategy with additional time between the 2 doses; this decision was not because of any adverse event. Each row represents a patient. The light blue sections are in proportion to the time from their presentation with MIS-C to their first dose of the BNT162b2 vaccine, and the white sections are in proportion to the time from their last vaccine until submission.

## Discussion

In this study, 15 patients treated for MIS-C after COVID-19 tolerated vaccination against SARS-CoV-2 without developing hyperinflammation, myocarditis, or reoccurrence of MIS-C up to 9.5 months after vaccination. This study provides critical information while the COVID-19 pandemic continues now that SARS-CoV-2 vaccination is available to children in the age range most at risk of developing MIS-C.^2^ Because of the rarity of MIS-C, our study is limited by a small sample size and its retrospective nature. However, with the known additive protection from reinfection provided by vaccinating previously infected individuals,^6^ these findings suggest that patients with a history of MIS-C can be offered vaccination against SARS-CoV-2.
